# Use of activated clotting time to monitor anticoagulation in patients receiving unfractionated heparin on renal replacement therapy

**DOI:** 10.1186/cc9542

**Published:** 2011-03-11

**Authors:** A Bidwai, R Sundaram

**Affiliations:** 1 RLUH, Liverpool, UK; 2 RAH, Glasgow, UK

## Introduction

The aim of our study was to determine the correlation between activated clotting time (ACT) and APTT values in patients receiving unfractionated heparin (UFH) for renal replacement therapy (RRT).

## Methods

A retrospective analysis was made of case notes and laboratory data of 39 critically ill patients who were on UFH for RRT over a 1-year period. There were 183 paired APTT and ACT measurements done at the same time (29 patients). APTT was done at the laboratory and ACT was done at the bedside using an ACTALYKE monitor (Array Medical). Target APTT and ACT ranges for UFH during RRT were 45 to 55 seconds (control 27 to 32 seconds) and 250 to 270 seconds (control 180 to 220 seconds). Datasets were divided into three groups and the correlation coefficient (Pearson's) was calculated using SPSS software.

## Results

**Figure 1:**
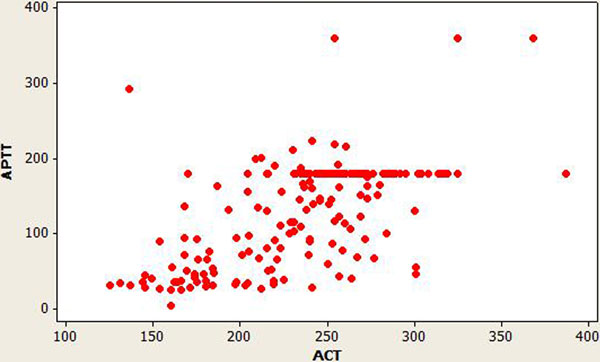
**Scatterplot of ACT versus APTT**.

Mean APTT was 129.5 ± 68.29 (range 25.6 to 360) seconds and mean ACT was 234.6 ± 47.02 (range 125 to 387) seconds. APTT and ACT values were divided into three datasets in a 3 × 3 table. There was no correlation between APTT and ACT values (kappa score being 0.12). There were more above-range APTT values (140/183) against above-range ACT values (36/183). See Table 1 and Figure 1.

**Table 1:** ACT versus APTT

	High ACT	Low	Normal
High APTT	35	70	36
Low	0	29	2
Normal	1	7	0

## Conclusions

Our data demonstrate that monitoring of anticoagulation with UFH using ACT cannot be recommended.
